# Biological Activities of Miracle Berry Supercritical Extracts as Metabolic Regulators in Chronic Diseases

**DOI:** 10.3390/ijms24086957

**Published:** 2023-04-09

**Authors:** Sonia Wagner, Marta Gómez de Cedrón, Joaquín Navarro del Hierro, Diego Martín-Hernández, María de las Nieves Siles, Susana Santoyo, Laura Jaime, Diana Martín, Tiziana Fornari, Ana Ramírez de Molina

**Affiliations:** 1 Precision Nutrition and Cancer Program, Molecular Oncology Group, IMDEA Food Institute, Universidad Autónoma de Madrid (CEI UAM + CSIC), 28049 Madrid, Spain; sonia.wagner@imdea.org; 2 Medicinal Gardens SL, Marqués de Urquijo 47, 28008 Madrid, Spain; 3 Institute of Food Science and Research (CIAL), Universidad Autónoma de Madrid (CEI UAM + CSIC), 28049 Madrid, Spain; joaqnava@ucm.es (J.N.d.H.); diego.martinh@uam.es (D.M.-H.); maria.siles@uam.es (M.d.l.N.S.); susana.santoyo@uam.es (S.S.); laura.jaime@uam.es (L.J.); diana.martin@uam.es (D.M.); tiziana.fornari@uam.es (T.F.); 4 Facultad de Veterinaria, Sección Departamental de Tecnología Alimentaria, Universidad Complutense de Madrid (ROR 02p0gd045), 28040 Madrid, Spain

**Keywords:** precision nutrition, bioactive extracts, miracle berry, colorectal cancer, lipid metabolism

## Abstract

*Synsepalum dulcificum* (*Richardella dulcifica*) is a berry fruit from West Africa with the ability to convert the sour taste into a sweet taste, and for this reason, the fruit is also known as the “miracle berry” (MB). The red and bright berry is rich in terpenoids. The fruit’s pulp and skin contain mainly phenolic compounds and flavonoids, which correlate with their antioxidant activity. Different polar extracts have been described to inhibit cell proliferation and transformation of cancer cell lines in vitro. In addition, MB has been shown to ameliorate insulin resistance in a preclinical model of diabetes induced by a chow diet enriched in fructose. Herein, we have compared the biological activities of three supercritical extracts obtained from the seed—a subproduct of the fruit—and one supercritical extract obtained from the pulp and the skin of MB. The four extracts have been characterized in terms of total polyphenols content. Moreover, the antioxidant, anti-inflammatory, hypo-lipidemic, and inhibition of colorectal cancer cell bioenergetics have been compared. Non-polar supercritical extracts from the seed are the ones with the highest effects on the inhibition of bioenergetic of colorectal (CRC) cancer cells. At the molecular level, the effects on cell bioenergetics seems to be related to the inhibition of main drivers of the de novo lipogenesis, such as the sterol regulatory element binding transcription factor (*SREBF1*) and downstream molecular targets fatty acid synthase (*FASN*) and stearoyl coenzyme desaturase 1 (*SCD1*). As metabolic reprograming is considered as one of the hallmarks of cancer, natural extracts from plants may provide complementary approaches in the treatment of cancer. Herein, for the first time, supercritical extracts from MB have been obtained, where the seed, a by-product of the fruit, seems to be rich in antitumor bioactive compounds. Based on these results, supercritical extracts from the seed merit further research to be proposed as co-adjuvants in the treatment of cancer.

## 1. Introduction

Miracle berry is a fruit from *Synsepalum dulcificum* (*Richardella dulcificum*), an indigenous shrub from tropical West Africa [1]. Recently, the EFSA (European Food Safety Authority) approved miracle berry (MB) as a novel food pursuant to Regulation (EU) 2015/2283 [2]. The fruit contains Miraculin, which is a member of the family of sweet proteins, such as thaumatin or brazzein, being responsible for transforming the sour taste into a sweet one [3]. The taste-modifying effect of the miracle fruit is effective under acidic conditions, and it lasts after 30–60 min approximately. Due to this ability, MB is used as a natural sweetener and for masking unpleasant flavors in the culinary area.

Further from the gastronomic point of view, MB can be interesting from a nutritional point of view due to the bioactive compounds present in the fruit. The pulp is rich in phenolic compounds (15.8%) and flavonoids (11.9%), while the skin contains higher amounts of phenolic compounds (36.7%) and flavonoids (51.9%), which have been associated with anti-oxidant activity [4].

In recent years, there has been an increased interest in using bioactive compounds and natural extracts from plants as co-adjuvants in the treatment of metabolic diseases including cancer. Cancer cells adapt their metabolism to support cell proliferation and dissemination. Together with the Warburg effect and the increased glutaminolysis, the altered lipid metabolism in cancer has been highlighted. Lipids not only provide building blocks to support cell proliferation, but they are also implicated in malignant transformation and dissemination. Moreover, many lipids metabolism-related enzymes have been proposed as biomarkers of cancer prognosis. Few studies have evaluated the antitumor activities of extracts from miracle berry (MB). Moreover, the effects of MB have been shown to ameliorate dysgeusia in cancer patients treated with chemotherapy [5], and its composition that is rich in flavonoids and other phenolic compounds [4] has been shown to have a high antioxidant capacity as a free radical scavenger and therefore can protect against free radical damage [6]. Nevertheless, only polar extracts from the fruit have been shown to inhibit cell proliferation and transformation in vitro [7,8].

Colorectal cancer (CRC) is the third most common cancer and the second leading cause of cancer-related deaths worldwide [9]. The main risk factors predisposing to colorectal cancer, in addition to genetic factors, include lifestyle factors such as red meat consumption, high consumption of sugars, and visceral obesity [10]. MB has been demonstrated to ameliorate insulin resistance in a preclinical model of prediabetes in rats [11] and to augment the uptake of glucose (GLUT4 transporter) in C2C12 myocytes [12]. The actual knowledge about the potential therapeutic applications of MB has been summarized in a previous review [13].

To expand the potential application of MB as a co-adjuvant in the treatment of CRC, herein, we have characterized the biological activities of four supercritical extracts—three obtained from the seed (S1, S2, S3), a subproduct of the fruit, and one obtained from the pulp and the skin (PS). The four supercritical extracts have been characterized in terms of total polyphenol (TPC) as well as other identified compounds of interest by gas chromatography–mass spectrometry, as pentacyclic triterpenes. Moreover, the antioxidant, anti-inflammatory, hypo-lipidemic activities, and the inhibition of cell viability and cell bioenergetics have been compared. The most apolar supercritical extracts from the seed (S1 and S2) are the ones with the highest effects on the inhibition of the bioenergetic metabolism of colorectal (CRC) cancer cells, which are the ones with reduced antioxidant activity. At the molecular level, inhibition of CRC cell viability seems to be related to the inhibition of main drivers of the de novo lipogenesis, such as the sterol regulatory element binding transcription factor (*SREBF1*) and downstream molecular targets such as fatty acid synthase (*FASN*) and stearoyl coenzyme desaturase 1 (*SCD1*). In addition, genes related to inflammation are downregulated. As metabolic reprograming is considered as one of the hallmarks of cancer, natural extracts from plants may provide complementary approaches in the treatment of cancer. Herein, for the first time, four supercritical extracts from MB have been obtained, where the ones obtained from the seed, a by-product of the fruit, seem to be rich in antitumor bioactive compounds. Based on these results, supercritical extracts from the seed merit further research to be proposed as co-adjuvants in the treatment of cancer.

## 2. Results

### 2.1. Extraction Yield

Supercritical fluid extraction (SFE) was applied to obtain four supercritical extracts using the grounded seeds (S) and lyophilized pulp and skins (PS) as the starting material. The conditions used to obtain the supercritical extracts are indicated in Table 1, together with the corresponding extraction yield, which was calculated as the weight of crude dried extract obtained with respect to the weight of S or PS used for the extraction (expressed as percentage).

As expected, the yield of extracts from the seed (S1, S2, and S3) increased with the amount of ethanol cosolvent used, from 3.48% to 4.80%. Furthermore, these yields were in the same order of magnitude of the yield obtained in the extraction of pulp and skins (PS). Although pulp and skins were also extracted with 0% and 14% ethanol cosolvent, these extracts were not studied due to the very low yield (lower than 0.5%) obtained in the absence of cosolvent and the precipitation of compounds and line obstruction produced in the SFE equipment when 14% ethanol cosolvent was used.

### 2.2. Chemical Composition of SFE Extracts

#### 2.2.1. Chemical Characterization by GC-MS

**Figure 1:**
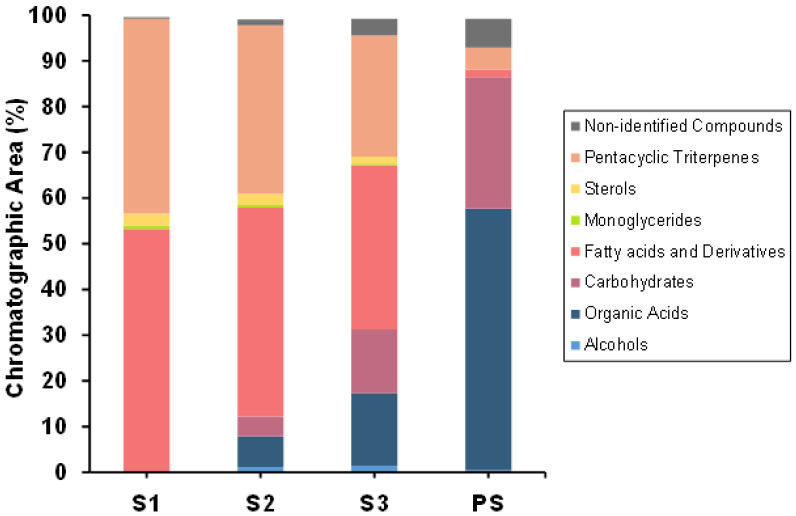
Chromatographic area (%) of each characterized family of compounds from S1, S2, S3, and PS supercritical extracts from MB.

The characterization of bioactive compounds in the different extracts was performed by GC-MS, where a total of 69 compounds were identified. These compounds were grouped into six main chemical families: alcohols, organic acids, lipids (fatty acids and derivates), carbohydrates (included monoglycerides), sterols, pentacyclic triterpenes, and others. Figure 1 illustrates the complex composition of the extracts and the total content of each major chemical group. The composition by chemical families is represented in units of chromatographic abundance and % of the area. The individual components are indicated in Supplementary Table S2.

**Figure 2:**
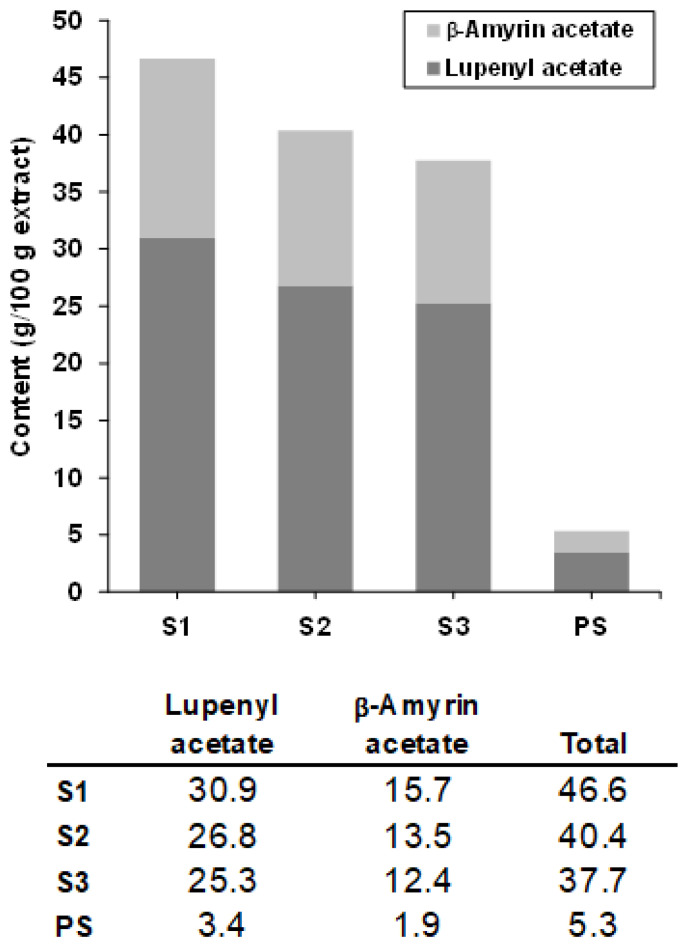
Content (g of compound/100 g of extract) of lupenyl acetate and b-amyrin acetate from S1, S2, S3, and PS supercritical extracts from MB.

Among all the compounds identified as potential bioactive molecules, the pentacyclic triterpenes “lupenyl acetate” and “β-amyrin acetate” stood out, especially in the case of the seed extracts. The pentacyclic triterpenoids belong to a class of C30 isoprenoids and were present in all parts of the plant [14]. Being in turn the compounds with the highest abundance of area, their quantification was carried out with corresponding commercial patterns, as shown in Figure 2.

Lupenyl acetate predominated in the seed extracts, being in the range of 25–31% of total extract, whereas β-amyrin acetate was in the range of 12–16%. The content of both pentacyclic triterpenes increased as the co-solvent decreased for the SFE extraction. The content of these compounds was minor for the PS extract.

Finally, among other chemical groups of interest, the chromatographic abundance of fatty acids and derivatives and organic acids was also remarkable. In the first case, linoleic acid, oleic acid, and palmitic acid stood out as the most abundant compounds within this group. These compounds were especially abundant for the seed extracts obtained in absence of co-solvent and were minor in case of the PS extract. On the contrary, the family of organic acids increased with the use of co-solvent during extraction for the seed extracts but was especially abundant in the case of the PS extract. Within this family, isocitric acid and malic acid were the most abundant organic acids.

#### 2.2.2. Determination of the Total Phenolic Content (TPC) and Individual Phenolic Compounds by HPLC-PAD

Phenolic compounds are secondary compounds of plant metabolism, commonly present in fruits and vegetables. It was found that the total phenolic content was very low for all the miracle berry extracts, irrespective of the different extraction conditions used (Table 2). HPLC-PAD identification of the individual content in phenolic compounds showed this poor content. However, in this case, noticeable differences were found regarding the use of pulp + skin instead of seeds from miracle berries. In that regard, all the seed extracts present only the same two compounds (based on their retention time), which show a spectrum compatible with a hydroxybenzoic acid structure, albeit different from those reported in the scientific literature for this berry [4,6,7,15,16].

Quantification of these compounds with a *p*-hydroxybenzoic acid analytical standard showed similar contents between the seed extracts, ranging from 0.15 to 0.18, and 4.89 to 6.82 mg/g extract for these two compounds.

PS extract, as expected, showed a different profile to that of the seed extracts. Although PS had a low content in phenolic compounds, it was possible to identify the presence of gallic acid and hyperoside, along with small concentrations of other not identified benzoic acids, flavonols, quercetin derivative, and anthocyanin derivative (Table 3).

### 2.3. Antioxidant Activity of SFE MB Extracts

#### 2.3.1. In Vitro Antioxidant Activity

There are many studies that associate the presence of phenolic compounds with antioxidant activity of the product. In the present study, the four supercritical extracts, as expected due to the low content in phenolics, hardly presented in vitro antioxidant activity measured by two different methodologies (Table 2).

#### 2.3.2. Cellular Antioxidant Activity

The cellular antioxidant activity was also determined. This activity measures the ability of a sample to inhibit the formation of reactive oxygen species (ROS). To do this, the method described in Wolfe and Liu [17] was followed in Caco-2 cells. The results obtained are shown in Table 4, and they are expressed as EC_50_ (μg/mL), which is the amount of extract needed to inhibit the production of reactive oxygen species by 50%. In general, the four extracts have either no or a very low antioxidant cellular activity. Therefore, this result seems to be in line with the low presence of phenolic compounds in the SFE extracts.

### 2.4. Effect of MB SFE Extracts on the Inhibition of Cell Viability of Colorectal Cancer Cells

**Figure 3:**
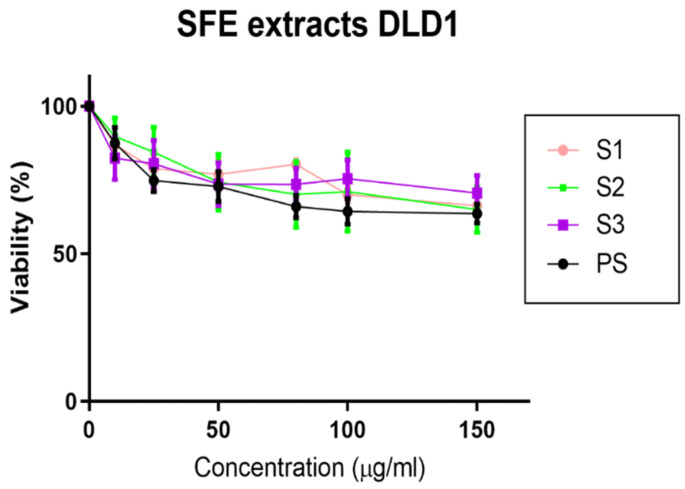
Effect of the supercritical extracts on cell viability (MTT assay) of DLD1 colorectal cancer cells. Dose–response curves of the cell proliferation assay after 48 h of treatment with increasing concentrations of MB SFE extracts. Data represent mean ± SEM of three independent experiments, each performed in triplicate.

It has been extensively described that lupeol and amyrine exert antiproliferative effects in cancer cell lines with a great potential in the prevention and treatment of cancers, including hepatocellular carcinoma [18], osteosarcoma [19], or colorectal cancer [20]. Due to large presence of lupeol and amyrine derivates in the seed extracts (S1, S2, and S3), we next wanted to evaluate the effects of the different extracts in the inhibition of cell viability of cancer cells. For this, DLD1 colorectal cancer cells were treated for 48 h with the different extracts. As shown in Figure 3, none of the extracts compromised DLD1 cell viability at the doses tested, given that the IC_50_ values were higher than 150 μg/mL, suggesting the absence of cytotoxicity at these doses. The effect of the extracts was also evaluated in the normal epithelial cell line CCD-841. While treatments at 150 μg/mL reduced DLD1 CRC cells’ viability in a 20–30%, the same doses did not affect cell viability of CCD-841 cells (Supplementary Figure S1). These results suggest that there is a therapeutic window for the use of MB supercritical extracts in the clinical practice.

### 2.5. Effect of MB SFE Extracts on the Bioenergetic Profile of Colorectal Cancer Cells

Cancer cells adapt their metabolism to support cell proliferation and dissemination. Thus, metabolic reprogramming has been highlighted as one of the hallmarks of cancer [21]. For this reason, we next wanted to investigate the effect of the four extracts on the bioenergetic profile of colorectal cancer cells. By means of the latest technology, we investigated the effects of the SFE extracts on the mitochondrial respiration of cancer cells. With this purpose, we monitored the oxygen consumption rates (OCRs) of CRC cells previously pre-treated with the extracts at two different doses, d1: 20 μg/mL and d2: 50 μg/mL, which were below the IC_50_ values (>100 μg/mL). Importantly, after 48 h of treatment, the very same number of cells that were plated in the absence of the extracts to compare the OCR were compared. On the day of the experiment, cells were exposed to a substrate-limited medium, with reduced availability of extracellular FAs (1% FBS) and glucose (0.5 mM Glucose). A total of 0.5 mM carnitine was added to favor the use of intracellular FAs for fatty acid oxidation (FAO).

**Figure 4:**
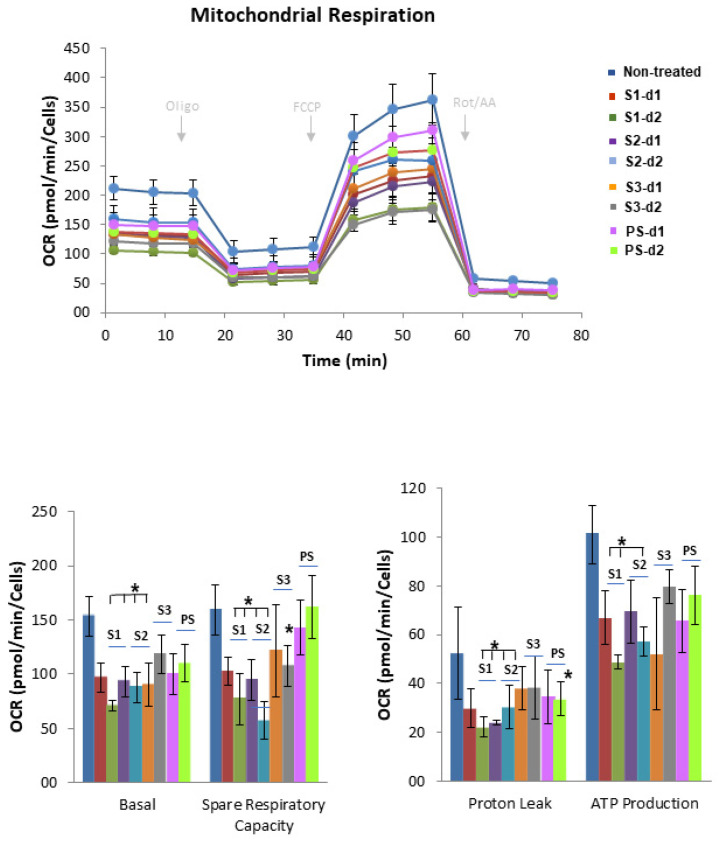
S1 and S2 diminish the mitochondrial oxidative phosphorylation. Mitochondrial respiration quantification by flux analysis of the oxygen consumption rate (OCR) of DLD1 cells previously pre-treated with SFE extracts for 48 h. The basal respiration rate, spare respiratory capacity, ATP production, and proton leak of 8000 cells per condition are compared. Data represent mean ± SEM of three independent experiments, each performed with four to six replicates. Asterisks * indicate *p* value < 0.05.

As it can be observed in Figure 4, the higher effects on the inhibition of the oxidative phosphorylation were found after S1 and S2 treatment. This result is very important, as it suggests that S1 and S2 are not able to maintain oxidative phosphorylation when there is a reduced availability of extracellular FAs and glucose. Interestingly, extracts S3 and PS did not present any effect on mitochondrial respiration, suggesting that SFE extracts obtained with the most apolar solvents may extract main bioactive compounds with the ability to inhibit cancer cell bioenergetics. Targeting the oxidative phosphorylation has been proposed to inhibit not only cancer cell proliferation but also dissemination, as reactive oxygen species (ROS) from mitochondria are drivers of the activation of prosurvival and the epithelial to mesenchymal transition (EMT) program in cancer.

As shown in Figure 4, S1 and S2 SFE extracts had a dose-dependent effect on the inhibition of the basal and maximal respiration rates of colorectal cancer cells. In line with these results, the calculated ATP levels were also diminished by S1 and S2.

As far as our knowledge, no previous studies have been performed in which the cell bioenergetics have been assessed with MB extracts.

### 2.6. Influence of MB SFE Extracts in the Expression of Lipid Metabolism Genes

**Figure 5:**
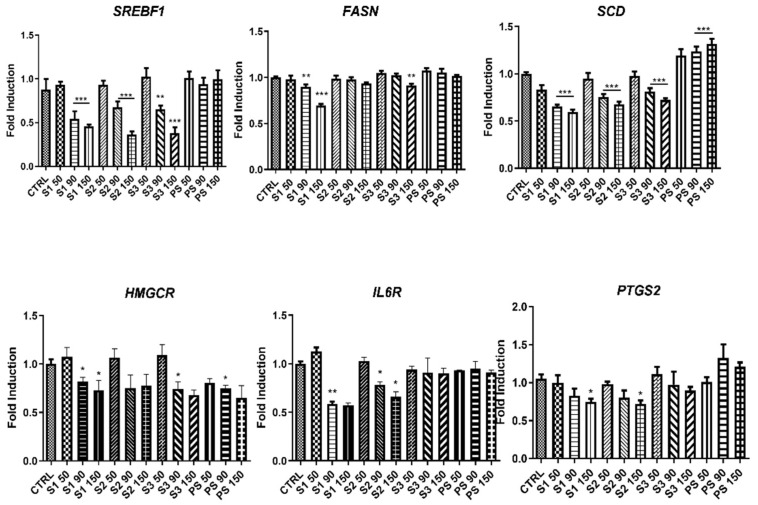
Seed-derived SFE extracts regulate lipid metabolic targets in the DLD1 CRC cell line. Asterisks indicate statistically significant differences (*p* < 0.05 (*); *p* < 0.01 (**); *p* < 0.005 (***)) relative to the control non-treated cells (3 replicates, three independent experiments).

Many authors have described alterations in the expression of lipid metabolism genes in cancer proliferation, dissemination, and prognosis [22,23]. As S1 and S2 diminished the oxidative phosphorylation in a medium with reduced availability of extracellular FAs and glucose, we next investigated the effect of the extracts on the main drivers of de novo lipogenesis and cholesteroigenesis, such as the sterol regulatory element binding transcription factor (*SREBF1*), and downstream molecular targets such as fatty acid synthase (*FASN*), stearoyl-CoA desaturase (*SCD*1), and hydroxy-methylglutaryl-CoA-reductase (*HMGCR*). SFE extracts S1 and S2 displayed the higher effects on the inhibition of the expression of the de novo lipogenesis, suggesting that these effects may be implicated on the inhibition of the mitochondrial oxidative phosphorylation of colorectal cancer cells (Figure 5).

In addition, the analysis of other mediators of lipid metabolism implicated in inflammation such as *PTGS2* and *ILR6* were significantly inhibited in the presence of S1 and S2.

### 2.7. Anti-Inflammatory Effect of MB SFE Extracts

As lipid metabolism mediators implicated on inflammation were found to be downregulated by S1 and S2 extracts, we next evaluated the effect of the extracts on THP-1/M inflammatory response after LPS activation.

First, extracts were evaluated for cytotoxicity on THP-1/M cells by the MTT method. Results showed that at 50 µg/mL, the higher concentration used in anti-inflammatory assays, no extract showed cytotoxicity (cell viability ≥ 95%).

**Figure 6:**
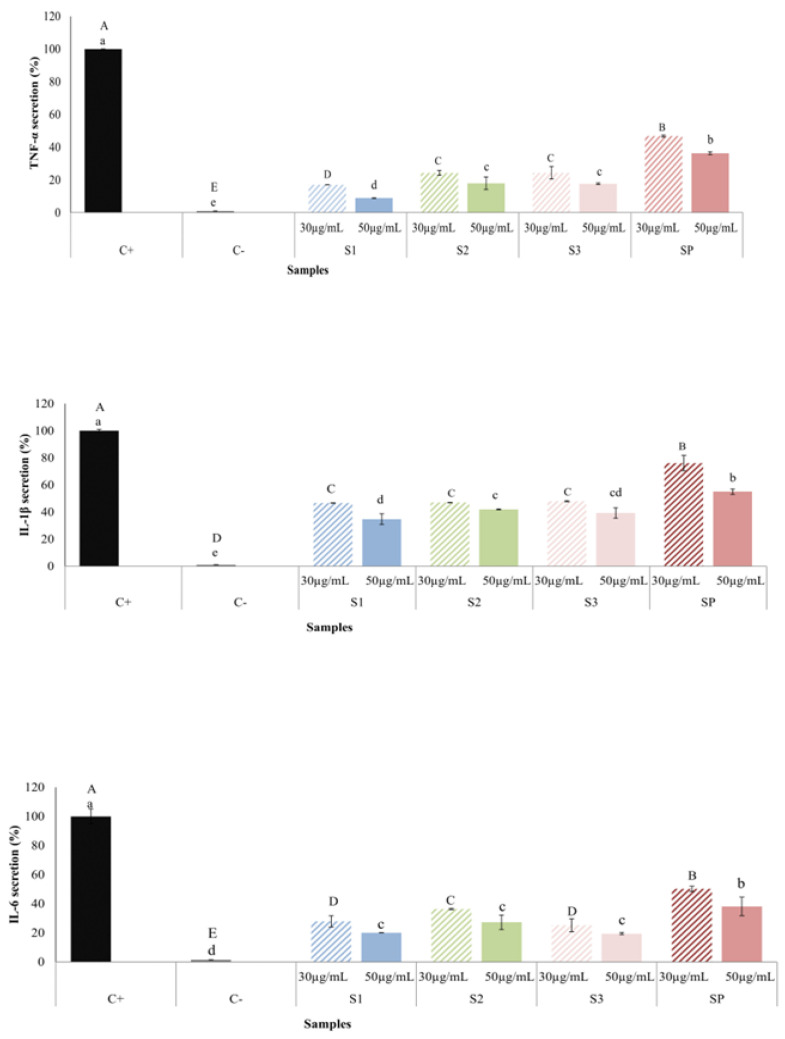
Levels of TNF-α, IL-1β, and IL-6 secreted by THP-1/M activated with LPS, in the presence of 30 or 50 μg/mL of MB SFE extracts for 24 h. Positive control (C+), LPS-stimulated cells. Negative control (C−), not LPS-stimulated cells. Each bar is the mean of three determinations ± S.D. Capital letters A, B, C, D and E show statistical differences between among miracle berry samples S1, S2, S3 and SP at 30 µg/mL, C+ and C−. Letters in lower case a, b, c, d and e show statistical differences among miracle berry samples S1,S2, S3 and PS at 50 µg/mL, compared to C+ and C−. Significance level at *p* < 0.05 according to Fisher’s least significant difference (LSD) test.

To evaluate the anti-inflammatory activity of extracts, LPS-activated THP-1/M were exposed to 30 and 50 µg/mL of SFE MB-extracts (S1, S2, S3, or PS). Figure 6 showed positive control (LPS-treated cells) presented an important increase in the release of the three studied proinflammatory cytokines, in contrast with negative control (non-stimulated cells). Regarding TNF-α secretion, the addition of 30 and 50 µg/mL of MB-extracts decreased the secreted level of this cytokine. At both concentrations tested, S1 and S2 extracts presented a higher inhibition of TNF-α secretion compared to PS extract, given that S1 the most active extract. At 30 µg/mL, S1 extract presented a secretion reduction of approximately 80%. The results obtained for IL-1β release in presence of extracts were similar to those obtained for TNF-α, since extracts from the seed (S1, S2, and S3) decreased the release of this cytokine by a greater percentage than the PS extract. Finally, seed-derived extracts also exhibited an important inhibition of IL-6, specially S1 and S2, with approximately 75% inhibition.

According to above results, all the extracts presented an important anti-inflammatory activity although extracts from the seeds (S1, S2, and S3) were more active than PS extract.

Figure 6 shows the levels of TNF-α, IL-1β, and IL-6 secreted by THP-1/M pre-activated with LPS, in the presence of 30 or 50 μg/mL of miracle berry extracts for 24 h.

### 2.8. Potential Hypolipidemic Effects of MB SFE Extracts

**Figure 7:**
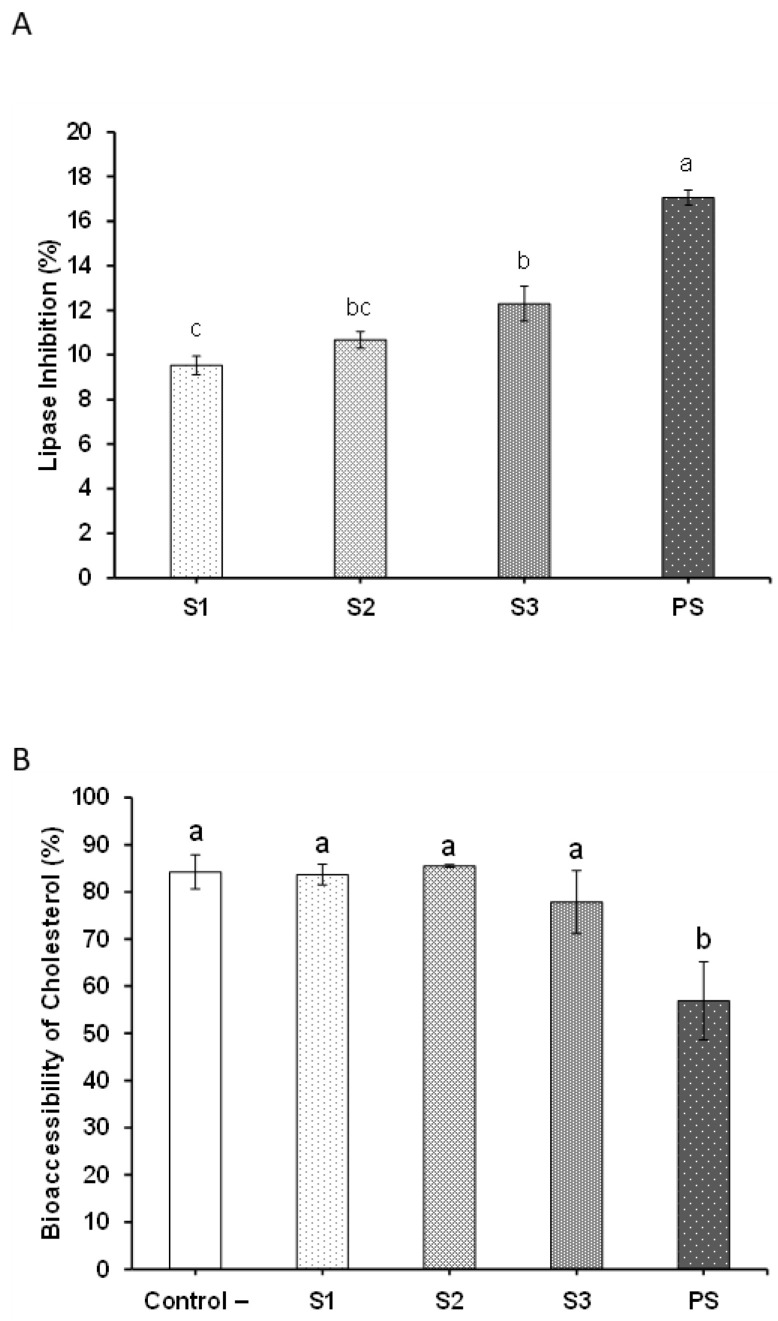
(**A**) Effect of S1, S2, S3, and PS extracts (at 0.75 mg/mL) on the inhibition of pancreatic lipase enzyme (%) under simulated intestinal conditions. (**B**) Bioaccessibility of cholesterol (%) after gastrointestinal digestion of cholesterol in absence (Control −) and presence of S1, S2, S3, and PS extracts at 1 mg/mL. Mean values with different letters (a–c) are significantly different if *p* ≤ 0.05.

In order to assess if the extracts could be potentially used as nutraceuticals to reduce the serum levels of lipids and cholesterol, we firstly assessed their ability to inhibit the pancreatic lipase in a simulated intestinal environment. The pancreatic lipase is an intestinal enzyme responsible for the hydrolysis of lipids from the diet and their further absorption. As shown in Figure 7A, the extracts were capable of inhibiting the pancreatic lipase, although in a very moderate manner. The concentrations assayed did not allow estimating the IC_50_, so the inhibition percentage is shown at the highest concentration studied (0.75 mg/mL). Among the SFE extracts obtained from the grounded seeds (S1, S2, and S3), the inhibitory activity seemed to significantly increase along with the amount of ethanol co-solvent used for producing such extracts (from 9.5% inhibitory activity to 12.3%). The extract produced from the pulp and skin (PS) showed the greatest inhibitory potential among the four extracts (17% inhibitory activity) (*p* < 0.001). This result was interesting considering that the composition of PS was considerably different compared to the extracts produced from the seed.

Regarding the potential hypocholesterolemic effect of the extracts, an in vitro gastrointestinal digestion model was employed in order to assess if the bioaccessibility of cholesterol was affected in the presence of the extracts. As shown in Figure 7B, none of the SFE seed extracts (S1, S2, and S3) were able to interfere with the bioaccessibility of cholesterol, as the fraction of bioaccessible cholesterol remained similar to that in absence of the extracts (around 80% bioaccessibility). However, PS extract did cause a significant effect on the bioaccessibility of cholesterol after the whole digestion process, as nearly 57% of cholesterol was bioaccessible. Thus, PS extract was capable of producing a 32% reduction in the bioaccessibility of cholesterol at a concentration of 1 mg/mL in the digestion medium.

## 3. Discussion

Colorectal cancer (CRC) is the third most common cancer globally with an annual rate of 10% of new cancer cases. It is the second deadliest cancer in the world, accounting for 9.4% of cancer related deaths [9]. Treatments—such as surgical resection of the tumor, chemotherapy, radiotherapy [24,25], and targeted immunotherapy [26]—depend on the stage and the molecular alterations in the tumors. Bioactive products from natural sources have been increasingly considered in the design of anti-cancer drugs due to their biological activities, as well as low toxicity and side effects. In addition, the use of natural bioactive extracts are of enormous interest as they may synergistically modulate several targets in cancer to overcome drug resistance [27]. In vitro and in vivo studies showed that consumption of natural products obtained from fruits and vegetables is also helpful in metastatic CRC [28].

Bioactive extracts from berries are of enormous interest due to the complex amount of useful phytochemicals they contain, including polyphenols, flavonoids, or phenolic acids [29].

Miracle berry (MB) (*Synsepalum dulcificum)* is an indigenous fruit whose small, ellipsoid, and bright red berries have been described to transform a sour taste into a sweet one. MB is rich in terpenoids, phenolic acids, and flavonoids, which are responsible for their described antioxidant activities. Most of the studies to evaluate the biological activities of MB-derived extracts have used berry’s flesh as the starting raw material after extraction with polar solvents. Thus, MB-derived polar extracts have been shown to inhibit cancer cell proliferation and malignant transformation in vitro [30], and to be anti-hypercholesterolemic [31], antioxidant and anti-hyperuricemic [32,33,34] in preclinical models.

Here, for the first time, supercritical fluid extraction (SFE) has been applied to obtain bioactive extracts from the seeds (S1, S2, and S3) and the pulp + skin (PS). SFE extraction is a green technology minimizing waste generation with little to no solvent residue left behind [35].

Very few studies have been conducted to evaluate the biological activities of extracts from MB. The majority of these studies have focused on the quantification of TPC of extracts obtained using polar solvents. Methanolic extracts from the berry flesh [6] have been partially characterized, demonstrating the presence of epicatechin, rutin, quercetin, myricetin, kaempferol, gallic, ferulic, syringic acid, three anthocyanins (delphinidin glucoside, cyanidin galactoside, and malvidin galactoside), three tocopherols (α-tocotrienol, α-, and γ-tocopherol), and lutein. In these studies, the TPC has been shown to correlate with the in vitro antioxidant activities [12]. On the contrary, our data indicate that SFE extracts, due to their low TPC, had very low antioxidant activities (Table 4). This is important due to the dual role of antioxidants in cancer. Although the relationship between oxidative stress, presence of free radicals, and cell damage has been widely described [36,37], in recent years, clinical trials have shown a lack of success in treatments with some antioxidants, with an even protective effect on tumor cells [38,39].

As metabolic reprogramming is a well-recognized hallmark of cancer [40], we next evaluated the effect of SFE extracts on the inhibition of colorectal cancer cell metabolism. Although we did not observe inhibition of cell proliferation at the doses tested (IC_50_ > 100 μg/mL), we found that SFE extracts from the seed—mainly S1 and S2—inhibited the mitochondrial respiration, ending up with a depletion of the levels of ATP (Figure 4). The characterization of bioactive compounds in the SFE extracts by GC-MS allowed us the identification of 69 compounds, which were grouped into six main chemical families: alcohols, organic acids, lipids (fatty acids and derivates), carbohydrates (included monoglycerides), sterols, pentacyclic triterpenes, and others. Among all the compounds, the pentacyclic triterpenes “lupenyl acetate” and “β-amyrin acetate” stood out, especially in the case of the seed extracts S1 and S2. These results suggest that pentacyclic triterpenes may be implicated on the inhibition of CRC cell metabolism. In line with this, lupeol, a precursor of lupenyl acetate, has been demonstrated to inhibit melanoma cell metabolism in vitro through various mechanisms including induction of differentiation [41], and the inhibition of mitogen-activated protein kinase p38 (MAPK) [42]. Additionally, β-amyrin and α-amyrin acetate have been demonstrated to reduce risk factors in CRC such as insulin resistance and visceral obesity in preclinical models of high-fat diet (HFD)-induced obesity [43,44].

Many authors have described alterations in the expression of lipid metabolism genes in cancer proliferation, dissemination, and prognosis [22,23,45,46].

For this reason, we investigated the effects of SFE extracts on main drivers of de novo lipogenesis and cholesteroigenesis, such as the sterol regulatory element binding transcription factor (*SREBF1*), and downstream molecular targets such as fatty acid synthase (*FASN*) and stearoyl-CoA desaturase (*SCD*1), and hydroxy-methylglutaryl-CoA-Reductase (*HMGCR*). Importantly, SFE extracts S1 and S2 displayed the higher effects on the inhibition of the expression of the de novo lipogenesis, suggesting that these effects may be implicated on the inhibition of the mitochondrial oxidative phosphorylation of colorectal cancer cells (Figure 5). In addition, the analysis of other mediators of lipid metabolism implicated in inflammation such as *PTGS2* and *ILR6* were significantly inhibited in the presence of S1 and S2. In line with this, the levels of TNF-α, IL-1β, and IL-6 secreted by THP-1/M pre-activated with LPS, were diminished in the presence of SFE extracts from the seeds.

High serum levels of cholesterol and lipids have been linked to an increased risk of developing certain cancers, such as colon or rectal, among others [47,48]. In order to assess if the SFE extracts could be potentially used as nutraceuticals to reduce the serum levels of lipids and cholesterol, we assessed their ability to inhibit the pancreatic lipase in a simulated intestinal environment. Although all the SFE extracts were able to inhibit the pancreatic lipase activity in a very moderate manner, the extract produced from the pulp and skin (PS) showed the greatest inhibitory potential among the four extracts (17% inhibitory activity) (*p* < 0.001). This result was interesting considering that the composition of PS was considerably different compared to the extracts produced from the seed. However, no significant correlations were found between the characterized compounds potentially responsible for the inhibition of the enzyme and the inhibitory activity of each of the extracts.

An in vitro gastrointestinal digestion model was used to evaluate the effect of the SFE extracts on the bioaccessibility of cholesterol. None of the SFE seed extracts (S1, S2, and S3) were capable of interfering with the bioaccessibility of cholesterol, as a fraction of bioaccessible cholesterol remained like that in absence of the extracts (around 80% bioaccessibility). However, PS extract did cause a significant effect on the bioaccessibility of cholesterol after the whole digestion process, as nearly 57% of cholesterol was bioaccessible. Thus, PS extract was capable of producing a 32% reduction in the bioaccessibility of cholesterol at a concentration of 1 mg/mL. These results are in line with ethanolic extracts from MB, which have been shown to exert potent anti-hypercholesterolemic activity [31].

In summary, the recently novel food miracle berry is gaining great interest, not only for its capacity to transform the sour taste into a sweet one, but also as a novel source of bioactive compounds from subproducts of the fruit such as the seeds. Herein, innovative biotechnological approaches, such as the green technology of supercritical fluids, have been applied to obtain supercritical extracts from the seed and the skin and the pulp. The biological activities, including antioxidant, anti-inflammatory, hypolipidemic, and anti-proliferative activities, have been compared. Interestingly, SFE from the seeds, mainly S1 and S2, are the ones with the highest effects on the inhibition of cell bioenergetics, which seems not to be related to the antioxidant activities. Lipid metabolism targets implicated in cancer progression and prognosis are significantly inhibited by S1 and S2, which are SFEs with the higher amounts of the pentacyclic triterpenes lupenyl acetate and β-amyrin acetate. Moreover, S1 and S2 reduce inflammation. On the contrary, SFE from PS reduced the lipase pancreatic activity and the bioaccesibility of cholesterol in an in vitro model of gastrointestinal digestion. These results encourage further studies to proposed SFE seed extracts as coadjuvants in the inhibition of cancer metabolism. In addition, a complete identification of main bioactive compounds of the SFE extracts should be performed to combine bioactive compounds from the seeds and from the pulp + skin, as the observed biological activities may synergistically inhibit CRC metabolism and risk factors in cancer such as obesity, insulin resistance, and hypercholesterolemia.

One of the main limitations to translate results from in vitro studies to in vivo clinical benefits is the reduced bioaccesibility and bioavailability of bioactive compounds after the gastrointestinal digestion. In a previous work, we have evaluated in two clinical studies a bioactive vehicle based on bioactive alkylglycerols (PCT/ES2017/070263) that improved not only solubility of bioactive compounds from rosemary supercritical extract (SFRE) but synergized with its antitumoral effects. Moreover, activation of protective innate immunity was found [49,50]. Our in vitro results with supercritical extracts from MB provide relevant information on novel bioactive extracts, which could be proposed to be formulated for preclinical and clinical trials.

## 4. Materials and Methods

### 4.1. Reagents and Cell Culture

Miracle berry pulp and skin powder and seeds, obtained by freeze-drying and grinding processes, were provided by Medicinal Gardens, batch number: MB0719. Lupeol acetate was purchased from Cymit Quimica S.L. (Barcelona, Spain). β-amyrin acetate and N,O-bis-(trimethylsilyl)trifluoroacetamide (BSTFA) were purchased from Merck (Darmstadt, Germany).

DLD1 (colorectal cancer cells) and CCD-841 cells (normal epithelial cells) were obtained from American Type Culture Collection (ATCC, Manassas, VA, USA) and cultured under standard conditions of temperature (37 °C), humidity (95%), and carbon dioxide (5%) in DMEM (DLD1) or EMEM (CCD-841) supplemented with 10% FBS, 2 mM glutamine, and 1% of antibiotic/antimycotic solution (containing 10,000 units/mL penicillin base, 10,000 g/mL streptomycin base, and 25,000 ng/m amphotericin B; Gibco, Grand Island, NY, USA).

### 4.2. Supercritical Fluid Extraction of MB Extracts

A supercritical CO_2_ extraction pilot plant equipment (Model Thar SF2000, Thar Technology, Pittsburgh, PA, USA) was used to obtain the supercritical extracts. A detailed description of the equipment can be found elsewhere [51]. The equipment has one extraction cell of 270 mL and two separators, each 500 mL capacity, with independent pressure and temperature control, together with a CO_2_ pump and another liquid pump for the co-solvent supply. The equipment includes a recirculation system, which allows the condensation of CO_2_ due to a chiller.

The extraction conditions were 20 MPa, 40 °C, 70 g/min CO_2_ for 180 min in the extractions without co-solvent (ethanol), and 90 min in the extractions with ethanol, introducing 140 g of grounded seed and 80 g of lyophilized pulp and skins in the extraction cell. The experimental conditions are summarized in Table 1. The separators were maintained at 40 °C, with a pressure of 50 MPa in the first separator and 1 MPa in the second separator. The extracts were collected in the first separator since typically the amount of material collected in the second separator was less than 1% of the amount collected in the first separator. In the co-solvent extractions, ratios of 7% and 14% of ethanol (% mass) mixed with CO_2_ were used.

### 4.3. Determination of Phenolic Compounds in the Supercritical Fluid Extracts from MB

Total phenolic compounds (TPC) have been determined following the colorimetric method proposed by Singleton et al. [52]. Results were expressed as mg of gallic acid equivalents (GAE) per gram of extract.

The individual phenolic compound identification, and their quantification, was carried out by HPLC-PAD as described by Villalva et al. [51]. A total of 20 μL of the samples, prepared at 20 mg/mL, were analyzed. Chromatographic signals were acquired at 280, 320, 360, and 520 nm. Samples were analyzed in triplicate.

### 4.4. Characterization of the Supercritical Fluid Extracts by Gas Chromatography–Mass Spectrometry (GC-MS)

The characterization of the extracts by GC-MS was carried out following the protocol described in Navarro del Hierro et al. [53] with modifications. Samples were first derivatized with BSTFA at 20 mg/mL by heating for 60 min at 75 °C, which allowed the formation of trimethylsilyl derivatives of all those less volatile compounds containing carboxyl or hydroxyl functional groups. After cooling at room temperature for 5 min, derivatized samples were taken to 10 mg/mL with hexane and analyzed in an Agilent 7890A GC-MS equipment (Agilent Technologies, Santa Clara, CA, USA). It comprised a split/splitless injector, G4513A autoinjector, an electronic pressure control, and a 5975C triple-axis mass spectrometer detector. An Agilent HP-5MS capillary column (30 m × 0.25 mm i.d., 0.25 µm phase thickness) was used and the carrier gas was helium at a flow of 2 mL/min. Sample injections (1 µL) were conducted in splitless mode. The injector temperature was 310 °C and the mass spectrometer ion source and interface temperatures were 230 and 280 °C, respectively. The temperature of the oven was initiated at 40 °C and immediately after increased at a rate of 3 °C/min to 150 °C, held for 10 min. After, temperature was increased at 15 °C/min to 310 °C and held for 15 min (total run time: 72.33 min). The mass spectra were obtained by electron ionization at 70 eV. The scanning speed was 1.6 scans/s in a mass range of *m*/*z* 30–700. Identification of compounds was performed by the NIST MS data library and by the mass spectra according to the literature. Quantification of the major identified compounds, namely the pentacyclic triterpenes lupeol acetate and β-amyrin acetate, was performed by calibration curves obtained from commercially available standards.

### 4.5. Measurement of the Antioxidant Activity of Supercritical Fluid Extracts from MB

Antioxidant activity was evaluated using the ABTS radical scavenging and the DPPH radical scavenging as described [52,53]. The assays were carried out at a concentration of 20 mg/mL (which was the highest concentration that allowed the total solubilization of the sample. The samples were left to react until the absorbance reached a steady state. The cellular antioxidant activity (CAA) was measured using the human colorectal adenocarcinoma cell line Caco-2 (ATCC, Manassas, VA, USA). Cells were cultured in DMEM (Dubelcco’s Modified Eagle’s Medium) supplemented with 10% FBS (fetal bovine serum), 100 U/mL penicillin, 100 mg/mL streptomycin, 1% nonessential amino acids, and 2 mM l-glutamine (Gibco, London, UK). The cytotoxicity of extracts was firstly evaluated in Caco-2 cells using MTT test [54]. Then, CAA was tested following the method described by Wolfe and Liu [17] with modifications. Caco-2 cells (1.5 × 10^5^ cell/mL) were seeded in 96-well plates, and after 48 h, the medium was removed and the cells were washed with PBS (phosphate-buffered solution). Afterward, cells were incubated with the extracts in subtoxic concentrations and 25 µM of fluorescent marker DCFH-DA (2′,7′-Dichlorofluorescin diacetate). After 1 h, the media was removed, the cells were washed 3× with PBS, and 600 µM of the free radical initiator ABAP (2′,2′-Azobis (2-methylpropionamidine) dihydrochloride) in HBSS (Hanks’ balanced salt solution) was added to each well. Fluorescence readings (Plate reader Cytation 5, Bioteck) were taken every 5 min for 1 h at excitation/emission wavelengths of 485/538 nm, for 13 cycles. The reduction of fluorescence was calculated using Equation (1):

(1) $$(\%\;inhibition)=\left(1-\frac{AUC\;sample}{AUC\;blank}\right)\times 100$$

The 50% of inhibition was established as the IC_50_ value.

The measurement of the antioxidant activity of the extracts, determined by both methodologies, was carried out at least in triplicate.

### 4.6. Analysis of the Immunomodulatory Activity of Supercritical Fluid Extracts from MB

The human monocyte THP-1 cells (ATCC) were cultured in RPMI 1640 medium supplemented with 10% fetal bovine serum (FBS), 100 U/mL penicillin, 100 mg/mL streptomycin, and 2 mM l-glutamine. Cells were seeded at a concentration of (5 × 10^5^ cells/mL) in a 24-well plate in the presence of 100 ng/mL phorbol 12-myristate 13-acetate (PMA) to induce their differentiation to macrophages (THP-1/M). Then, differentiated cells were maintained for 48 h at 37 °C under 5% CO_2_ in a humidified incubator.

First, the MTT protocol was carried out to evaluate the miracle berry extracts’ cytotoxicity (S1, S2, S3, and PS) in differentiated macrophages. Afterwards, macrophages were washed with serum-free RPMI and then replaced with serum-free medium containing LPS (0.05 μg/mL) and subtoxic concentrations of the different supercritical extracts. After 24 h of incubation, cells supernatants were collected and stored at −20 °C.

Pro-inflammatory cytokines TNF-α, IL-1β, and IL-6 were quantified in the collected supernatants by BD Biosciences Human ELISA set (BD Biosciences, Aalst, Belgium) following the manufacturer’s instructions. The colored reaction was carried out by measuring the OD at 450 nm with substrate correction at 570 nm using a multiscanner autoreader (InfiniteM200, Tecan, Barcelona, Spain). THP-1/M stimulated with LPS and in the absence of extracts were used as positive control and considered as 100% cytokine secretion. The assays were conducted in three independent experiments, in triplicated wells.

### 4.7. Analysis of the Inhibition of Cell Viability of Supercritical Fluid Extracts from MB

DLD1 and CCD 841 cells were cultured in 96-well plates at densities of 5000 cells/well and 7500 cells/well overnight to allow the cells to attach. The next day, cells were treated for 48 h with the different MB SFE extracts solved in EtOH/Chloroform (2:1) diluted in DMEM (Cultek) growth medium at different concentrations. Subsequently, the culture medium was supplemented with 10% sterile filtered MTT at 5 mg/mL. After 3 h, the medium was removed, and the insoluble formazan crystals were dissolved in 100 µL/well of DMSO. Absorbance was measured at 560 nm in a Victor Nivo multimode plate reader (Perkin Elmer). The inhibition of growth (mitochondrial function) due to extracts was expressed as a percentage of viable cells in experimental wells relative to control wells, therefore calculating IC_50_ values. Three independent experiments were performed with each of the extracts.

### 4.8. Gene Expression Analysis

Total RNA was extracted with standard protocol using TriReagent (Sigma-Aldrich, Madrid, Spain). A total of 1 µg of RNA was reverse-transcribed with the high-capacity RNA-to-cDNA Master Mix system (Life Technologies, Carlsbad, CA, USA). Quantitative polymerase chain reaction (qPCR) was performed in the Quant Studio PCR System (Life Technologies, Carlsbad, CA, USA) using the Veri Quest SYBR Green qPCR Master Mix, and oligos were used (Supplementary Table S1). The 2^−ΔΔ^Ct method was applied to calculate the relative gene expression [55]. Beta-2-Microglobulin (*B2M*) gene was used as endogenous control.

### 4.9. Analysis of Mitochondrial Respiration by Extracellular Flux Analysis of the Oxygen Consumption Rate (OCR)

To analyze the mitochondrial respiration, we monitored the extracellular flux of the oxygen consumption rate (OCR) (Cell MitoStress Test) after the injection of several modulators of the electron transport chain, with the XFe96 Cell Bionalyzer (Xfe96, Seahorse Biosciences, Billerica, MA, USA). Optimal cell density and drugs titration were previously determined. Prior to the experiments, cells were pre-treated with indicated doses of the extracts for 48 h. Non-treated cells were kept as controls.

For MitoStress assay, DLD1 cells were seeded onto 96-well Xfe96 cell culture microplates at a density of 8000 cells/well. The next day, medium was changed to substrate-limited medium DMEM without glucose, glutamine, sodium pyruvate (Life Technologies), that was supplemented with 0.5 mM Glucose, 1.0 mM GlutaMAX (Life Technologies), 0.5 mM Carnitine (Sigma-Aldrich, Madrid, Spain), and 1% FBS (fetal bovine serum) for 6–8 h. Then, media changed to Xfe DMEM supplemented with 2.5 mM glucose, 0.5 mM carnitine, and 5 mM HEPES and adjusted to pH 7.4.

Cells were incubated for 45 min–1 h at 37 °C without CO_2_. Three different modulators of mitochondrial respiration were sequentially injected. After basal oxygen consumption rate (OCR) determination, oligomycin (1.5 µM), which inhibits ATPase, was injected to determine the amount of oxygen dedicated to ATP production by mitochondria. To determine the maximal respiration rate or spare respiratory capacity, FCCP (carbonyl cyanide-4-(trifluoro-methoxy) phenyl-hydrazone) was injected (0.8 µM) to free the gradient of H^+^ from the mitochondrial intermembrane space and thus to activate maximal respiration. Finally, antimycin A and rotenone (0.5 µM) were added to completely inhibit the mitochondrial respiration. The analysis of mitochondrial oxidative phosphorylation was performed with 5 replicates per plate in 3 independent experiments.

### 4.10. Hypolipidemic Activity

#### 4.10.1. Pancreatic Lipase Inhibition Assay

The inhibitory activity of the extracts against pancreatic lipase enzyme was measured by using 4-MUO as substrate under simulated in vitro intestinal conditions, as described by Herrera et al. (2019) [56]. A digestion solution consisting of 100 mM Trizma-Maleic buffer (pH 7.5, 0.15 M NaCl, 5.1 mM CaCl_2_), bile salts (7.8 mg/mL), and lecithin (3.12 mg/mL) was prepared to simulate the intestinal environment. The reaction mixture consisted of 0.5 mL of extract solution (S1, S2, S3, or PS) in digestion buffer, 0.5 mL of freshly prepared pancreatic lipase at 1 mg/mL (10 mg of lipase in 10 mL of digestion buffer, stirred for 10 min and centrifuged at 4000 rpm for 10 min), and 1 mL of 4-MUO solution at 0.1 mM in digestion buffer. Extracts were tested at different concentrations and prepared in triplicate. Control samples were prepared in the absence of extracts following the same procedure, and controls of extracts at the different concentrations were prepared in absence of lipase and substrate. Both types of controls were prepared in triplicate. Samples were placed in an orbital incubator protected from light (Titramax 1000 package, Heidolph Instruments, Schwabach, Germany) at 37 °C and 250 rpm for 20 min. Then, three aliquots of 0.150 mL were added to a 96-well plate, and the amount of 4-MUO hydrolyzed by lipase was measured using a fluorescence microplate reader (Polarstar Galaxy, BMG Labtechnologies, Offenburg, Germany), setting an excitation wavelength of 350 ± 10 nm and an emission wavelength of 450 nm. The inhibition of pancreatic lipase activity was calculated as follows:

$$Lipase\;Inhibition(\%)=100-\left[\left(\frac{F_{extract\;sample}-F_{extract\;control}}{F_{control\;sample}}\right)\times 100\right]$$

#### 4.10.2. Effect on the Intestinal Bioaccessibility of Cholesterol

An in vitro digestion model was applied to study the intestinal bioaccessibility of cholesterol, which was based on Navarro del Hierro et al. (2021) [57]. The whole process of digestion was performed in 50 mL tubes. First, a mixture containing 3 mg of lecithin, 8 mg of cholesterol, 80 mg of olive oil, and 5 mg of extract (S1, S2, S2, or PS) was prepared. A negative control (in absence of extracts) was also prepared. For the gastric digestion, 2.2 mL of a gastric solution (150 mM NaCl, 6 mM CaCl_2_, and 0.1 mM HCl, pH 2.5) and 0.450 mL of a fresh extract of gastric enzymes (16.7 mg/mL of gastric lipase and 29.4 mg/mL of pepsin) was added to the first mixture, which was stirred in an orbital incubator (Titramax 1000 package, 177 Heidolph Instruments, Heildelberg, Germany) at 37 °C and 250 rpm for 45 min. After 45 min of gastric digestion, 1.9 mL of a solution simulating a biliary secretion were added (50 mg of lecithin and 125 mg of bile salts in 0.25 mL of 350 mM CaCl_2_ solution, 0.75 mL of 3.25 M NaCl solution, and 5 mL of trizma-maleate buffer 100 mM pH 7.5, stirred for 10 min). The mixture was added to the digestion medium and stirred for 2 min at 37 °C and 190 rpm to allow the dispersion of the components. Then, the intestinal digestion was initiated by the addition of 0.450 mL of a fresh pancreatic extract at 166.7 mg/mL in trizma-base buffer, which had been previously stirred for 10 min and centrifuged for 15 min at 4000 rpm. Intestinal digestion was performed at 190 rpm for 60 min. The final concentration of the extracts in the digestion medium was 1 mg/mL. The digestion of each sample was prepared at least in duplicate.

In order to further determine the bioaccessibility of cholesterol, the digestion medium was submitted to centrifugation for 40 min at 4000 rpm. After centrifugation, 3 mL of the micellar phase, which contained the solubilized cholesterol, were collected and extracted with ethyl acetate at a ratio of 1:1 (*v*/*v*). The mixture was vortexed for 5 min and centrifuged at 3500 rpm for 5 min. The top phase was collected and directly analyzed on an LC-2030C 3D Plus system (Shimadzu, Kyoto, Japan) to quantify cholesterol, which was carried out on an ACE 3 C18-AR column (150 mm × 4.6 mm, 3 µm particle size) protected by a guard column (Avantor, Radnor, PA, USA). Isocratic flow was employed using methanol with 0.05% water as mobile phase. The flow rate was constant at 1.2 mL/min, and the column temperature was kept at 35 °C. The injection volume was 10 µL. UV-Visible spectra were recorded from 190 to 800 nm and the chromatograms were registered at 205 nm. Quantification of cholesterol was performed by calibration curve obtained from its corresponding commercial standard. Bioaccessibility of cholesterol in each of the samples was calculated as follows:

$$Bioaccessibility\;of\;cholesterol\;(\%)=\left(\frac{mg\;of\;cholesterol\;in\;micellar\;phase}{mg\;of\;cholesterol\;in\;digestion\;meidum}\right)\times 100$$

Any significant reduction in the bioaccessible cholesterol compared to the negative control was considered a potential hypocholesterolemic effect.

### 4.11. Data and Statistical Analysis

One-way analysis of variance (ANOVA; Bonferroni post-hoc test) was used to determine qPCR differences in gene expression. * *p* < 0.05, ** *p* < 0.01, *** *p* < 0.005, and **** *p* < 0.001 indicate statistic significant differences. GraphPad Prim 8.0.1 statistical software. was used for all statistical analyses. Results are expressed as mean ± standard deviation.

Statistical analysis was conducted using Statgraphics v. Centurion XVI for Windows (Statpoint Inc. Warranton, VA, USA). Results were expressed as mean ± standard deviation. A one-way analysis of variance (ANOVA) followed by Fisher’s least significant difference (LSD) test at *p* < 0.05 was used to look for difference among means.

One-way analysis of variance (ANOVA; Tukey’s post-hoc test) was used to determine hypolipidemic activity by means of the general linear model procedure of the SPSS 26.0 statistical package (SPSS Inc., Chicago, IL, USA). Results are expressed as mean ± standard deviation. Differences were considered significant at *p* ≤ 0.05.

## Figures and Tables

**Table 1:** Yield and conditions to obtain the supercritical extracts from the seed (S1, S2, and S3) and the pulp and skin (PS) from MB.

Extract	Source	Pressure (Bar)	T (°C)	CO_2_ (g/min)	EtOH (%)	Yield (%)
S1	Seed	200	40	70	0	3.48
S2	Seed	200	40	70	7	4.20
S3	Seed	200	40	70	14	4.80
PS	Pulp + skin	200	40	70	7	4.35

**Table 2:** Total phenolic content (TPC) and in vitro antioxidant activity of the SFE extracts from MB.

Extract	TPC(mg GAE/g Extract)	ABTS (mmol Trolox/g Extract)	DPPH (mmol Trolox/g Extract)
S1	13.70 ± 0.75	0.006 ± 0.000	0.000 ± 0.000
S2	14.33 ± 0.57	0.029 ± 0.032	0.005 ± 0.000
S3	16.43 ± 0.10	0.033 ± 0.001	0.009 ± 0.000
PS	11.83 ± 0.28	0.046 ± 0.002	0.021 ± 0.001

**Table 3:** Quantification of total phenolic compounds by HPLC-PAD in PS supercritical extract from the pulp and skin of MB.

Extract	Rt (min)	Compound	mg Compound/g Extract
PS	10.019	Gallic acid	0.05 ± 0.04
	10.739	Gallic acid isomer	0.14 ± 0.00
	11.995	Benzoic acid n.i.	0.02 ± 0.01
	12.123	Benzoic acid n.i.	0.04 ± 0.00
	15.556	Flavonol n.i.	1.52 ± 1.21
	17.476	Flavonol n.i.	1.46 ± 1.15
	17.675	Anthocyanin n.i.	0.17 ± 0.11
	18.226	Flavonol n.i.	2.06 ± 1.19
	18.521	Flavonol n.i.	1.55 ± 1.20
	22.372	Quercetin derivative.	0.02 ± 0.02
	22.771	Hyperoxide	0.02 ± 0.05
	28.645	Caffeic acid derivative	0.20 ± 0.10
	33.656	Flavonol n.i.	0.05 ± 0.01

**Table 4:** Cellular antioxidant activity of SFE extracts from MB.

Extract	EC_50_ (μg/mL)
S1	244.69 ± 5.70
S2	>2000
S3	1092.33 ± 5.31
PS	303.53 ± 3.88

## Data Availability

No applicable.

## Supplementary Materials

The following supporting information can be downloaded at: https://www.mdpi.com/article/10.3390/ijms24086957/s1.
