# Supplementary material for: Biological Activities of Miracle Berry Supercritical Extracts as Metabolic Regulators in Chronic Diseases
Source: Int J Mol Sci. 2023 Apr 9;24(8):6957. doi: 10.3390/ijms24086957 (PMC10138767; doi:10.3390/ijms24086957)
Supplement: Supplementary file 1 [file ijms-24-06957-s001.zip › ijms-2307304-supplementary.pdf]

Supplementary Table S1. Primers used for quantitative real-time PCR.

Gene Name	Forward prime	Reverse prime
SREBF1	CCGCCGCGCCTTGAC	AGCATAGGGTGGGTCAAATAGG
FASN	TATGAAGCCATCGTGGACGG	GAAGAAGGAGAGCCGGTTGG
SCD	TGCCCACCACAAGTTTTCAG	CATCAGCAAGCCAGGTTTGT
HMGCR	TGATTGACCTTTCCAGAGCAAG	CTAAAATTGCCATTCCACGAGC
PTSG2	ATCACAGGCTTCCATTGACC	CAGGATACAGCTCCACAGCA
IL6R	CCCCTCAGCAATGTTGTTTGT	CTCCGGGACTGCTAACTGG
GAPDH	TGGTATCGTGGAAGGACTCATGAC	ATGCCAGTGAGCTTCCCGTTCAGC

Supplementary Table S2. Identified compounds and their Chromatograms

S1-SFE

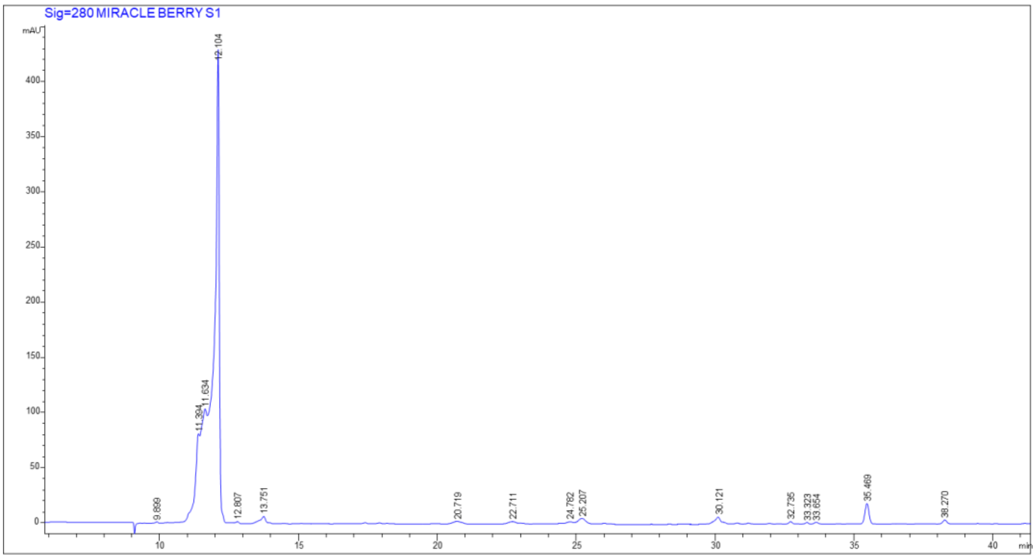

Retention Time	compound	mg compound/mg extract
12,104	Benzoic acid n.i	5,18 ± 0,12
35,469	Benzoic acid n.i	0,15 ± 0,03

S2-SFE

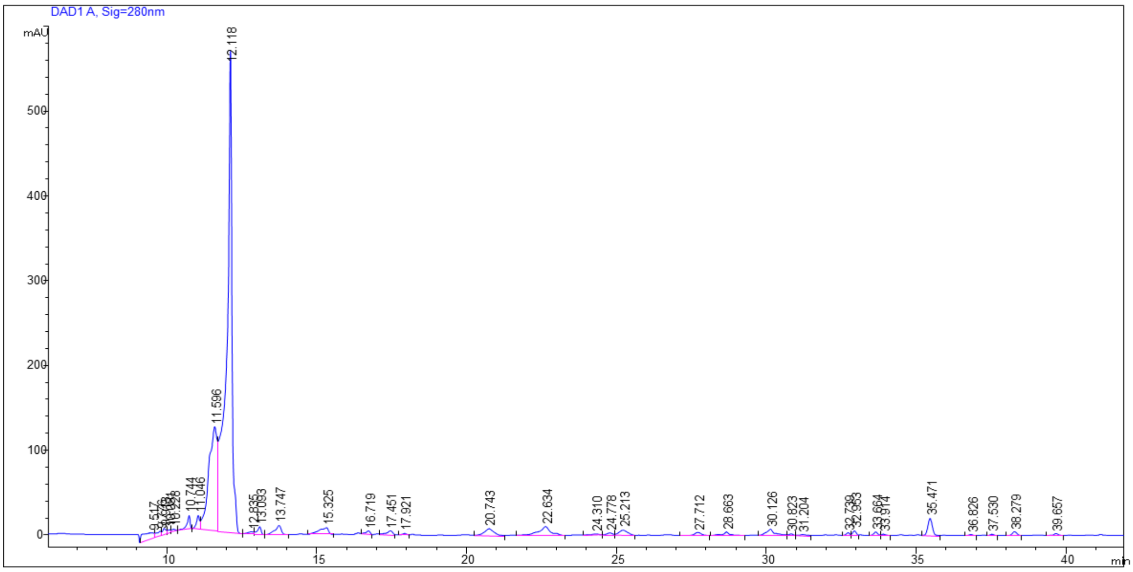

Retention Time	compound	mg compound/mg extract
12,118	Benzoic acid n.i	6,82 ± 0,03
35,471	Benzoic acid n.i	0,18 ± 0,01

S3-SFE

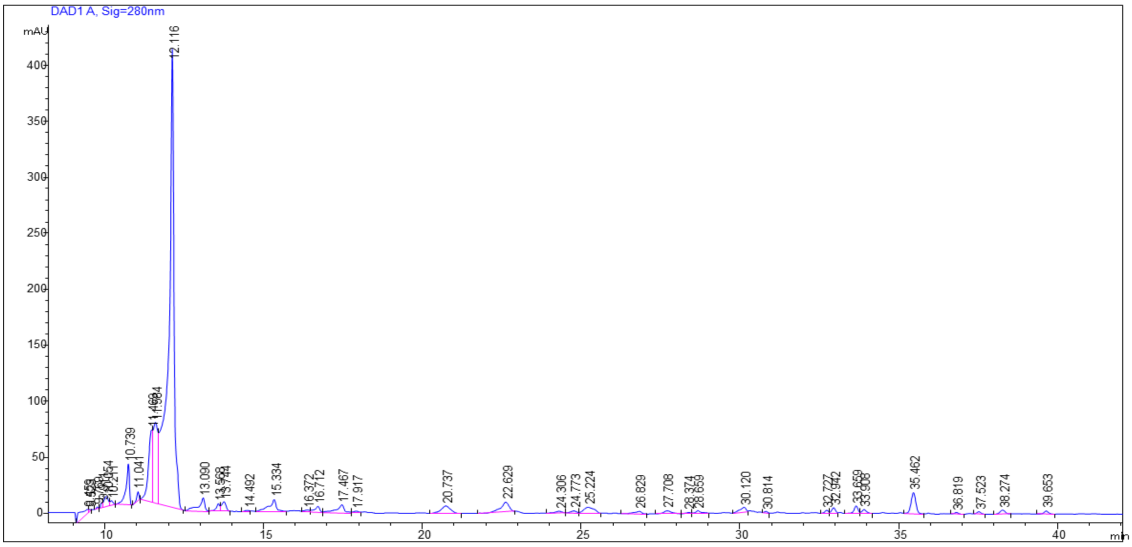

Retention Time	compound	mg compound/mg extract
12,116	Benzoic acid n.i	4,89 ± 0,26
35,462	Benzoic acid n.i	0,16 ± 0,03

PS-SFE

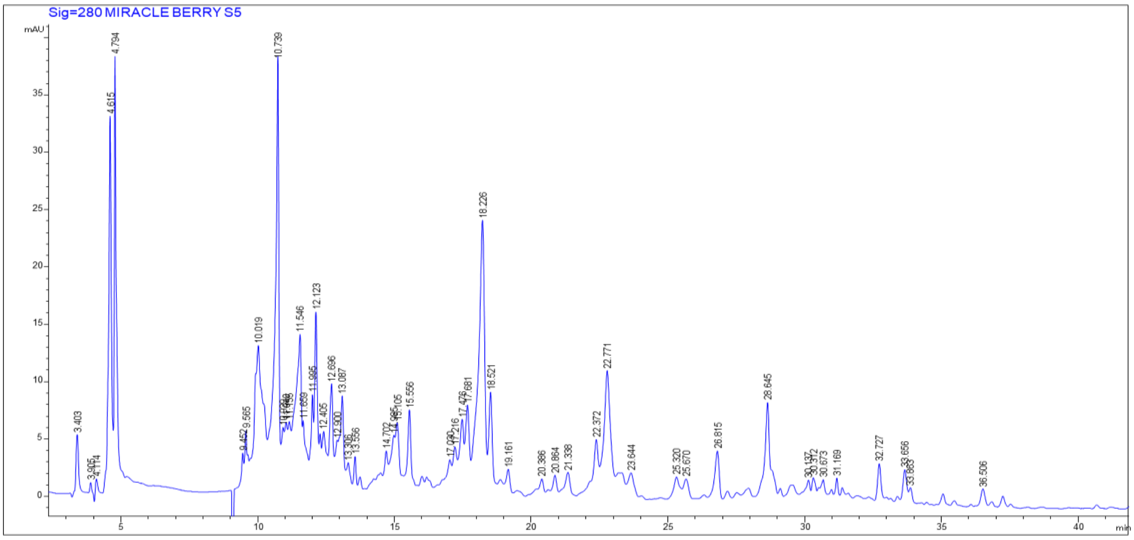

Retention Time	compound	mg compound/mg extract
10,019	Galic acid	0,05 ± 0,04
10,739	Galic acid n.i.	0,14 ± 0,00
11,995	Benzoic acid n.i.	0,02 ± 0,01
12,123	Benzoic acid n.i.	0,04 ± 0,00
15,556	Flavanol n.i.	1,52 ± 1,21
17,476	Flavanol n.i.	1,46 ± 1,15
17,675	Antocianin n.i.	0,17 ± 0,11
18,226	Flavanol n.i.	2,06 ± 1,19
18,521	Flavanol n.i.	1,55 ± 1,20
21,338	Cumaric acid n.i.	<LOQ*
22,372	Quercetin n.i.	0,02 ± 0,02
22,771	Hyperoside	0,02 ± 0,05
28,645	Rosmarinic acid	0,20 ± 0,10
33,656	Flavonol n.i.	0,05 ± 0,01

Supplementary Figure S1

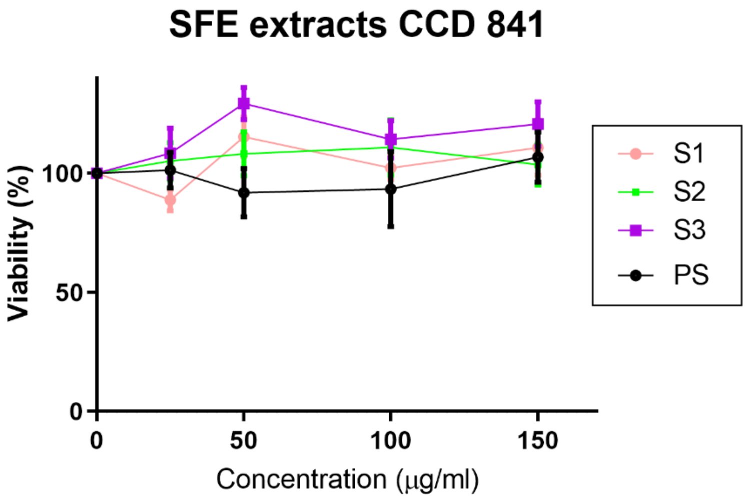

Effect of the supercritical extracts from MB on cell viability (MTT assay) of CCD 841 normal epithelial cells. Dose–response curves of the cell proliferation assay after 48 h of treatment

with increasing concentrations of MB SFE extracts. Data represent mean ± SEM of three independent experiments, each performed in triplicate
